# PET Imaging of VMAT2 with the Novel Radioligand [^18^F]FE-DTBZ-d4 in Nonhuman Primates: Comparison with [^11^C]DTBZ and [^18^F]FE-DTBZ

**DOI:** 10.1021/acschemneuro.1c00651

**Published:** 2021-11-23

**Authors:** Sangram Nag, Mahabuba Jahan, Miklós Tóth, Ryuji Nakao, Andrea Varrone, Christer Halldin

**Affiliations:** Department of Clinical Neuroscience, Center for Psychiatry Research, Karolinska Institutet and Stockholm County Council, Stockholm 17176, Sweden

**Keywords:** PET, VMAT2, radioligands, nonhuman primate, imaging, kinetics

## Abstract

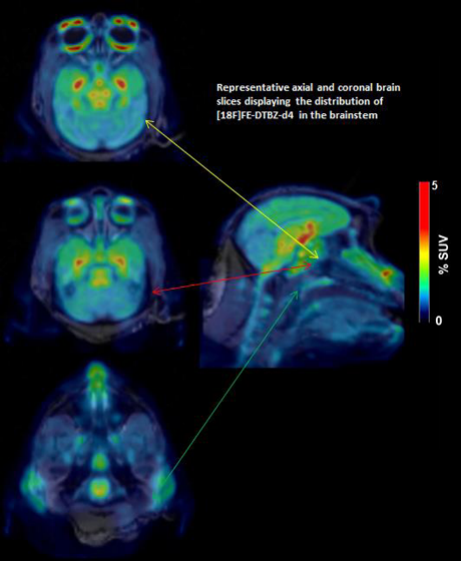

The vesicular monoamine transporter type 2 (VMAT2) is believed to be responsible for the uptake of monoamines into the vesicles of the synaptic terminals. Two VMAT2 radioligands [^11^C]DTBZ and [^18^F]FP-DTBZ have been used to assess the degree of nigrostriatal deficit in Parkinson’s disease (PD) using positron emission tomography (PET). [^18^F]FE-DTBZ-d4, the nondeuterated analogue of [^18^F]FE-DTBZ showed similar imaging properties with better stability against defluorination. Therefore, [^18^F]FE-DTBZ-d4 draws attention to be investigated as an imaging marker for VMAT2 in the brain. The aim of this study was to investigate the brain kinetics and quantification of [^18^F]FE-DTBZ-d4 in nonhuman primates (NHPs), with comparison to [^11^C]DTBZ and [^18^F]FE-DTBZ. Radiolabeling was successfully achieved either by one-step ^11^C-methylation or by a two-step fluorine-18 nucleophilic substitution reaction. The stability and radiochemical yield were analyzed with high-performance liquid chromatography (HPLC). Three female cynomolgus monkeys were included in the study and underwent a total of 12 positron emission tomography (PET) measurements. Each monkey was examined with each tracer. In addition, two pretreatment and one displacement PET measurements with tetrabenazine (2.0 mg/kg) were performed for [^18^F]FE-DTBZ-d4. All PET measurements were conducted using a high-resolution research tomograph (HRRT) system. Radiometabolites were measured in monkey plasma using gradient radio-HPLC. [^18^F]FE-DTBZ-d4 (SUV: 4.28 ± 1.01) displayed higher brain uptake compared to both [^18^F]FE-DTBZ (SUV: 3.43 ± 0.54) and [^11^C]DTBZ (SUV: 3.06 ± 0.32) and faster washout. Binding potential (BP_ND_) values of [^18^F]FE-DTBZ-d4 in different brain regions (putamen: 5.5 ± 1.4; caudate: 4.4 ± 1.1; midbrain: 1.4 ± 0.4) were higher than those of [^11^C]DTBZ and [^18^F]FE-DTBZ. [^18^F]FE-DTBZ showed faster radiometabolism in plasma compared to [^11^C]DTBZ and [^18^F]FE-DTBZ-d4. [^18^F]FE-DTBZ-d4 is a suitable radioligand for quantification of VMAT2 in the nonhuman primate brain, with better imaging properties than [^11^C]DTBZ and [^18^F]FE-DTBZ. A preliminary comparison suggests that [^18^F]FE-DTBZ-d4 has increased stability against defluorination compared to the nondeuterated analogue.

## Introduction

The vesicular monoamine transporter 2 (VMAT2) is an integral membrane protein, previously known as the synaptic vesicular monoamine transporter, mainly present in neuronal cells of the central, peripheral, and enteric nervous system.^1^ VMAT2 transports monoamines such as dopamine, norepinephrine, serotonin, and histamine from cellular cytosol into synaptic vesicles.^2^ VMAT2 is also required for the vesicular release of the neurotransmitter gamma-aminobutyric acid (GABA) in nigrostriatal and mesolimbic dopamine neurons.^3^

One of the most common neurodegenerative movement disorder, Parkinson’s disease (PD), is clinically characterized by akinesia, resting tremor, and rigidity. The degeneration of the dopaminergic neurons of the substantia nigra pars compacta (SNc) in PD leads to the loss of nigrostriatal terminals and to the reduction of dopamine levels in the striatum.^4,5^ There are several evidence that link VMAT2 to dopaminergic cell loss in PD. Previous studies have reported that the dysfunction of VMAT2 can evoke cytoplasmic dopamine accumulation, which leads to dopaminergic neuron death.^6^ Other studies have shown that protein expression levels of VMAT2 were significantly reduced in PD patients^7^ and that an increased VMAT2 level or function might protect against the development of PD.^8,9^ Therefore, striatal VMAT2 is considered as a presynaptic marker of dopamine terminal loss in PD.^10^

Several dihydrotetrabenazine (DTBZ) derivatives have been labeled with ^11^C/^18^F and developed as PET radioligands. Affinity of dihydrotetrabenazine (DTBZ) toward VMAT2 binding is stereospecific,^11^ and the (+)-enantiomer showed 1000-fold better binding affinity (*K_i_* = 0.97 ± 0.48 nM) over the (−)-enantiomer (*K_i_* = 2.2 ± 0.3 μM).^12,13^ Therefore, PET measurements have been performed mostly with the (+)-enantiomer of DTBZ derivatives. Two VMAT2 radioligands, [^11^C]-(+)-DTBZ^14,15^ and [^18^F]FP-(+)-DTBZ ([^18^F]AV-133),^16−18^ have already been used in several studies in patients with PD. ^18^F-fluoroethyl-(+)-dihydrotetrabenazine (^18^F-FE-DTBZ) has not been evaluated in humans, but a previous study in rats showed a relatively poor striatum-to-cerebellum ratio.^19^

VMAT2 is a protein that is also expressed in insulin-producing beta cells in pancreatic islets and might serve as a potential marker for the assessment of the beta cell mass.^20^ VMAT2-specific [^11^C]-(+)-DTBZ has been shown to be a beta cell mass (BCM) biomarker with potential to distinguish between healthy and diabetic subjects longitudinally, however, with some difficulties such as less affinity for VMAT2 and higher fat solubility, which translates into high nonspecific binding.^21−23^ In addition, ^18^F has a longer half-life than ^11^C, making it suitable for a wider range of applications. In an effort to develop a ^18^F-labeled VMAT2 radioligand for imaging the beta cell mass, our group investigated [^18^F]FE-(+)-DTBZ in a large piglet model.^24^ The radioligand was found to be metabolized extensively by *in vivo* defluorination. To improve the *in vivo* stability, a deuterated analogue [^18^F]FE-(+)-DTBZ-d4 of [^18^F]FE-DTBZ^25^ has been recently developed and investigated in pigs for *in vivo* imaging of VMAT2 in insulin-secreting beta cells. PET/CT studies showed that [^18^F]FE-(+)-DTBZ-d4 had lower bone uptake than [^18^F]FE-(+)-DTBZ. This property is of advantage for brain imaging, considering that bone uptake might interfere with the measurement of specific binding of ^18^F-radioligands in the central nervous system (CNS).^26^ For this reason, [^18^F]FE-(+)-DTBZ-d4 was considered a potential imaging marker of VMAT2 in the brain. The in vivo properties of [^18^F]FE-(+)-DTBZ-d4 remain, however, to be investigated.

The current study was, therefore, designed to investigate the brain uptake, the kinetic properties, the radiometabolism, and the noninvasive quantification of [^18^F]FE-(+)-DTBZ-d4 and compared with [^11^C]-(+)-DTBZ and [^18^F]FE-(+)-DTBZ, in nonhuman primates (NHP).

## Results and Discussion

[^11^C]DTBZ was synthesized in a one-step via *O*-methylation reaction where [^11^C]CH_3_OTf was used as a methylating agent. The incorporation yields of [^11^C]CH_3_OTf into [^11^C]-DTBZ were high (80–90%) for all productions. The total time of the radiosynthesis was 28–30 min, and the range of the molar activity (MA) was >160 GBq/μmol at the end of the synthesis (EOS). The radiochemical purity was more than 98% for all productions at the EOS. The formulated solution of all three radioligands was found to be radiochemically stable for up to 60 min and the radiochemical purity was >98%.

The radiosynthesis of [^18^F]FE-DTBZ-d4 and [^18^F]FE-DTBZ was accomplished via the two steps. ^18^F-fluoroethylation reaction: The alkylating agents, [^18^F]FEtBr-d4 and [^18^F]FEtBr, were generated from 2-bromo-1,2-tetra-^2^H-ethyl tosylate and bromoethyl tosylate, respectively, by a one-step nucleophilic ^18^F-fluorination reaction as described previously.^27^ The purified [^18^F]FEtBr-d4 or [^18^F]FEtBr was trapped in the basic solution of the precursor in *N,N*-dimethylformamide (DMF) and heated at 110 °C for 5 min. After HPLC purification, 1.0–1.7 GBq [^18^F]FE-DTBZ-d4 or [^18^F]FE-DTBZ was obtained; total synthesis time was 100 ± 10 min. The average radiochemical yield of the radiosynthesis was 9% (nondecay corrected). The identity of [^18^F]FE-DTBZ-d4 or [^18^F]FE-DTBZ was confirmed by coinjection with authentic reference onto the radio-HPLC. The radiochemical purity was >98% and molar activity (MA) was >100 GBq/μmol at the time of administration.

**Figure 1:**
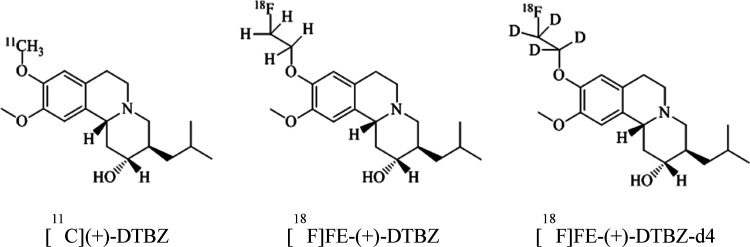
Structures of [^11^C]-(+)-DTBZ, [^18^F]FE-(+)-DTBZ, and [^18^F]FE-(+)-DTBZ-d4.

**Figure 2:**
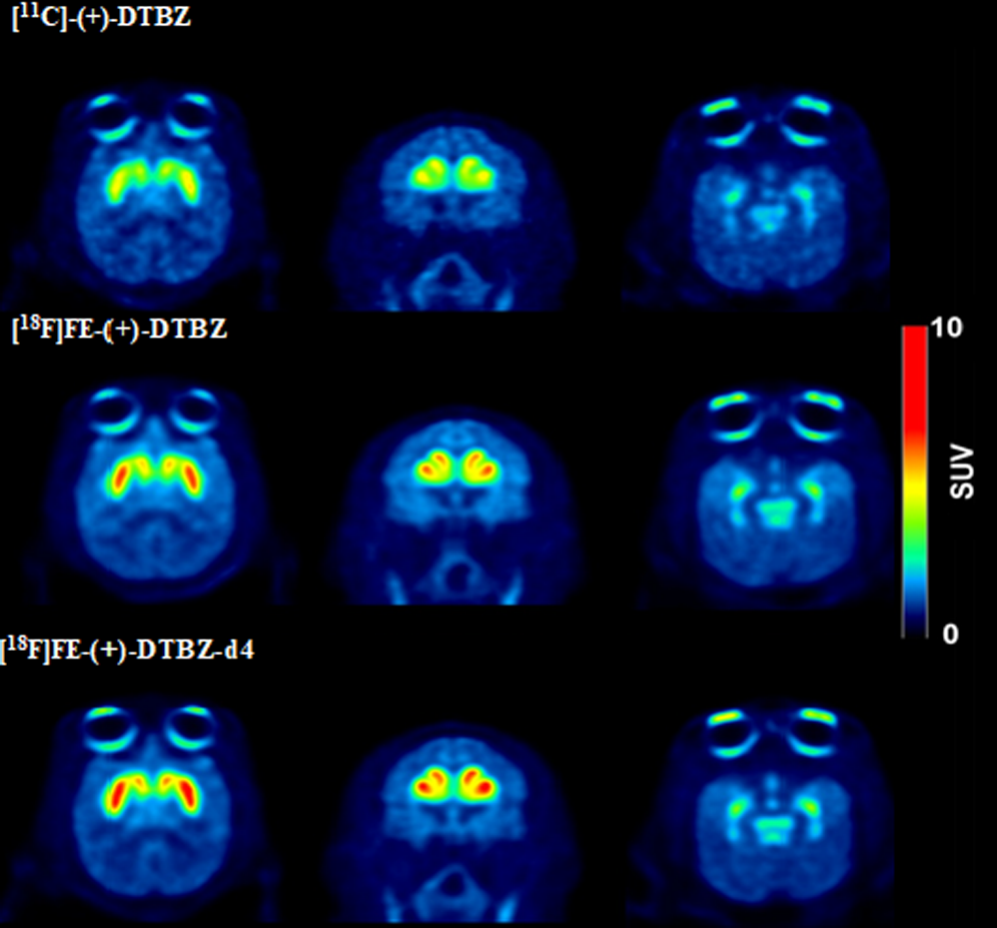
Representative fused PET images of [^11^C](+)-DTBZ, [^18^F]FE-(+)-DTBZ, and [^18^F]FE-(+)-DTBZ-d4.

**Figure 3:**
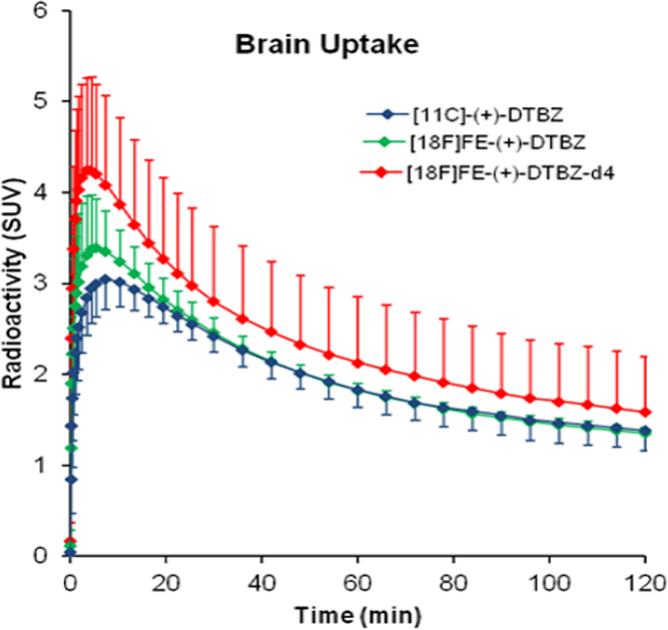
Relative SUV time–activity curves (TACs) of [^11^C]-(+)-DTBZ, [^18^F]FE-(+)-DTBZ, and [^18^F]FE-(+)-DTBZ-d4 at baseline conditions.

**Figure 4:**
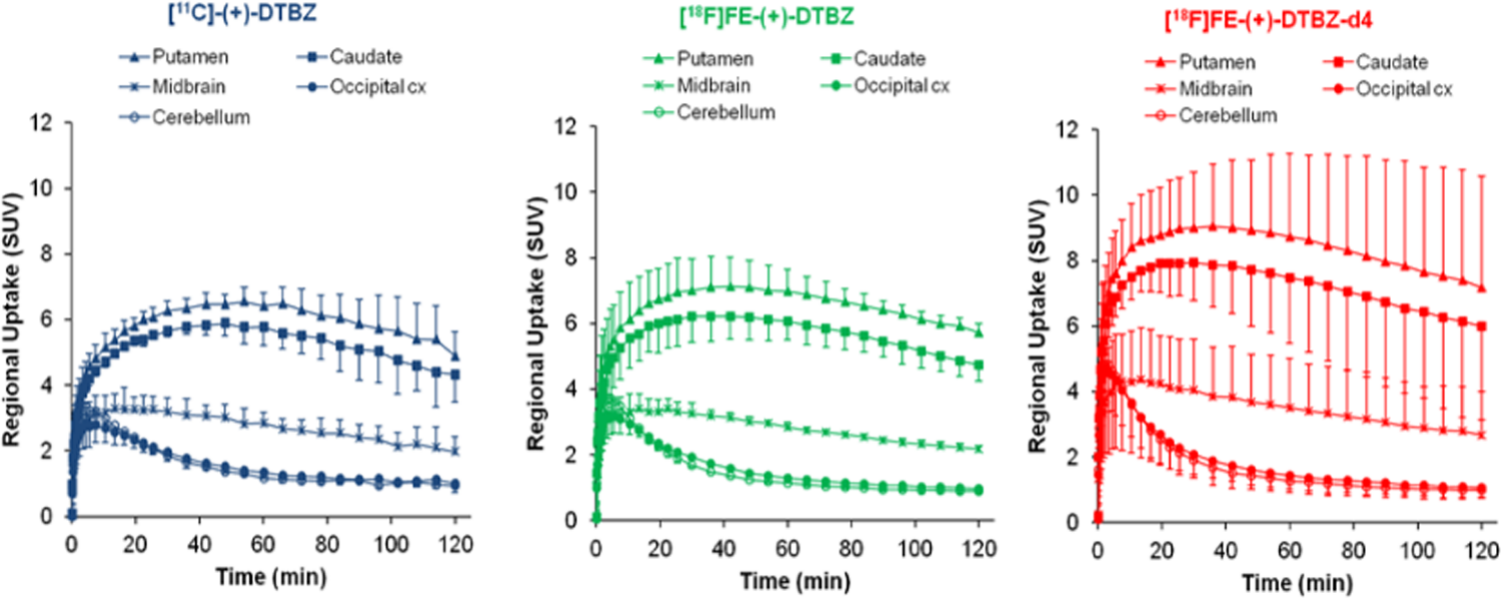
Regional brain uptake of [^11^C]-(+)-DTBZ, [^18^F]FE-(+)-DTBZ, and [^18^F]FE-(+)-DTBZ-d4.

Three cynomolgus monkeys were studied with [^11^C]-(+)-DTBZ, [^18^F]FE-(+)-DTBZ, and [^18^F]FE-(+)-DTBZ-d4 (Figure 1). The injected radioactivity, injected mass, and the MA at the time of injection are shown in Table 1. Fusion images of MRI and summated PET (average between 9 to 123 min) of NHP2 are shown in Figure 2. The time–activity curves (TAC) of [^11^C]-(+)-DTBZ, [^18^F]FE-(+)-DTBZ-d4, and [^18^F]FE-(+)-DTBZ uptakes in a cynomolgus monkey brain are shown in Figure 3. All three radioligands cross the blood–brain barrier and bind rapidly with a time to peak on average of 9 ± 3, 6 ± 2, and 4 ± 2 min for [^11^C] (+)-DTBZ, [^18^F]FE-(+)-DTBZ, and [^18^F]FE-(+)-DTBZ-d4, respectively. [^18^F]FE-DTBZ-d4 displayed the highest brain uptake (SUV: 4.28 ± 1.01%) compared to [^18^F]FE-DTBZ (SUV: 3.43 ± 0.54) and [^11^C]DTBZ (SUV: 3.06 ± 0.32%) and also the highest peak-to-120 min ratio (3.4 ± 0.5) in comparison to the other two radioligands (Table 2). The highest radioactivity concentration was observed in putamen followed by caudate and midbrain; the lowest uptake was observed in the cerebellum for all three radioligands, which is in accordance with the literature (Figure 4). Binding potentials (BP_ND_) were calculated by Logan’s noninvasive analysis. BP_ND_ values of [^18^F]FE-DTBZ-d4 (putamen: 5.5 ± 1.4; caudate: 4.4 ± 1.1; midbrain: 1.4 ± 0.4) were higher than those of [^18^F]FE-DTBZ (putamen: 4.7 ± 0.3; caudate: 3.8 ± 0.5; midbrain: 1.1 ± 0.1) and [^11^C]DTBZ (putamen: 3.7 ± 0.6, *p* = 0.07; caudate: 3.0 ± 0.6, *p* = 0.04; midbrain: 0.9 ± 0.3, *p* = 0.02). [^18^F]FE-(+)-DTBZ-d4 showed slightly earlier peak equilibrium (40 min) as compared with [^11^C]-(+)-DTBZ and [^18^F]FE-(+)-DTBZ (>60 min).

**Table 1:** Body Weight of NHPs, Injected Radioactivity, Mass, and the MA at the Time of Injection

parameters	[^11^C](+)-DTBZ	[^18^F]FE-(+)-DTBZ	[^18^F]FE-(+)-DTBZ-d4
body weight (kg)	6.5 ± 0.6	6.7 ± 0.3	6.5 ± 0.6
injected radioactivity (MBq)	181 ± 7	186 ± 10	187 ± 14
MA (GBq/μmol)	410 ± 418	465 ± 206	254 ± 26
injected mass (μg)	0.2 ± 0.2	0.2 ± 0.1	0.3 ± 0.0

**Table 2:** Whole Brain Uptake and Time to Peak of [^11^C]-(+)-DTBZ, [^18^F]FE-(+)-DTBZ, and [^18^F]FE-(+)-DTBZ-d4

parameter	[^11^C]-(+)-DTBZ	[^18^F]FE-(+)-DTBZ	[^18^F]FE-(+)-DTBZ-d4
time to peak (min)	9 ± 3	6 ± 2	4 ± 2
peak SUV%	3.06 ± 0.32	3.43 ± 0.54	4.28 ± 1.01
ratio peak-to-120 min	2.3 ± 0.5	2.5 ± 0.5	3.4 ± 0.5

**Figure 5:**
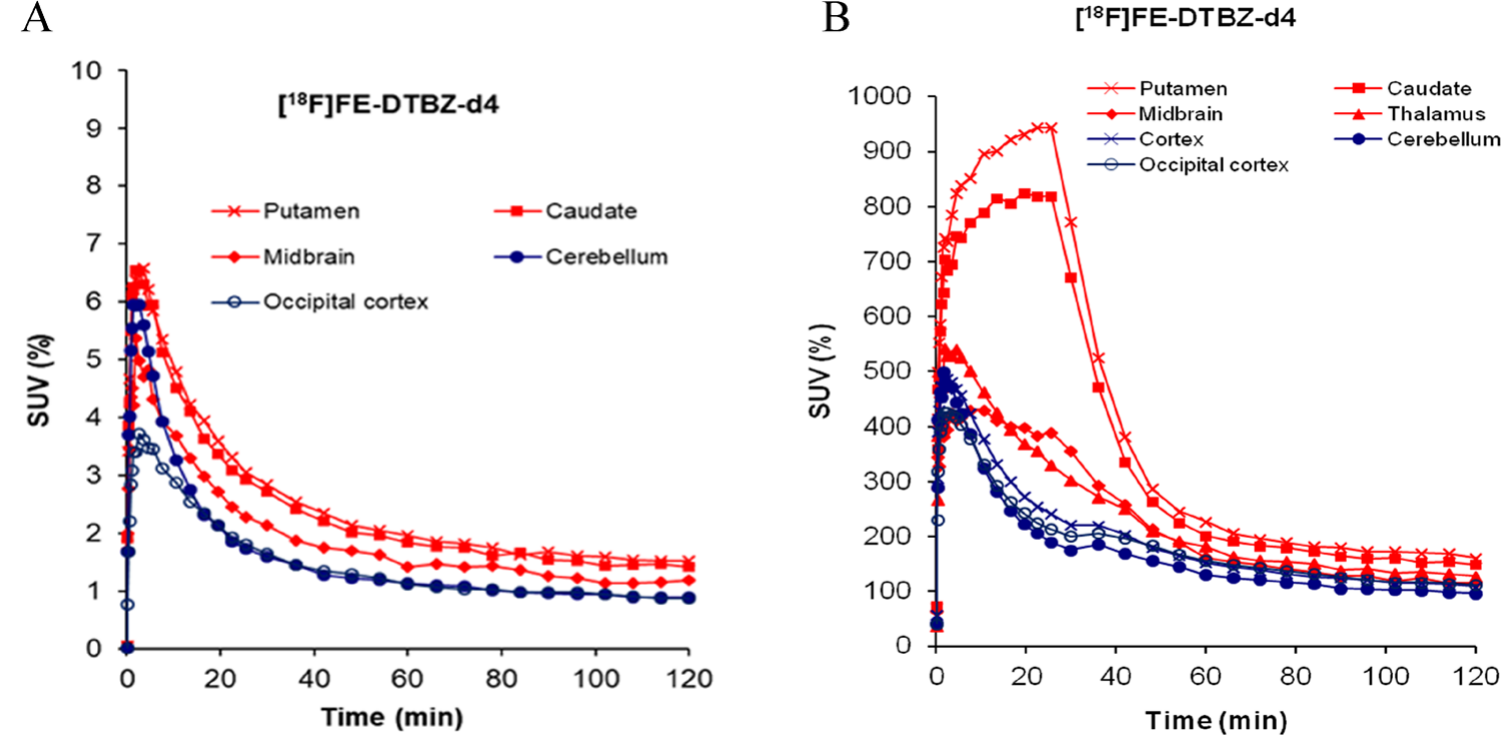
Regional brain uptake of [^18^F]FE-(+)-DTBZ-d4. (A) Pretreatment with tetrabenazine (2 mg/kg) 15 min prior to the administration of [^18^F]FE-DTBZ-d4. (B) Displacement experiment by administration of tetrabenazine (2 mg/kg) 25 min after the injection of [^18^F]FE-DTBZ-d4.

Since [^18^F]FE-DTBZ-d4 showed higher uptake in all brain regions of NHPs, further investigations were accomplished to observe specific VMAT2 binding uptake by performing two pretreatment and one displacement experiments using the VMAT2-specific compound tetrabenazine. After pretreatment with tetrabenazine (2 mg/kg, 15 min prior to radioligand injection), the uptake of [^18^F]FE-DTBZ-d4 in caudate, putamen, and midbrain was decreased almost to the level of the cerebellum and occipital cortex (Figure 5A). During the displacement experiment, the administration of tetrabenazine (2 mg/kg, 25 min. after radioligand injection) determined a clear reduction of [^18^F]FE-DTBZ-d4 uptake in caudate, putamen, and midbrain, almost to the level of the cerebellum and occipital cortex (Figure 5B), demonstrating the reversibility of the binding.

**Figure 6:**
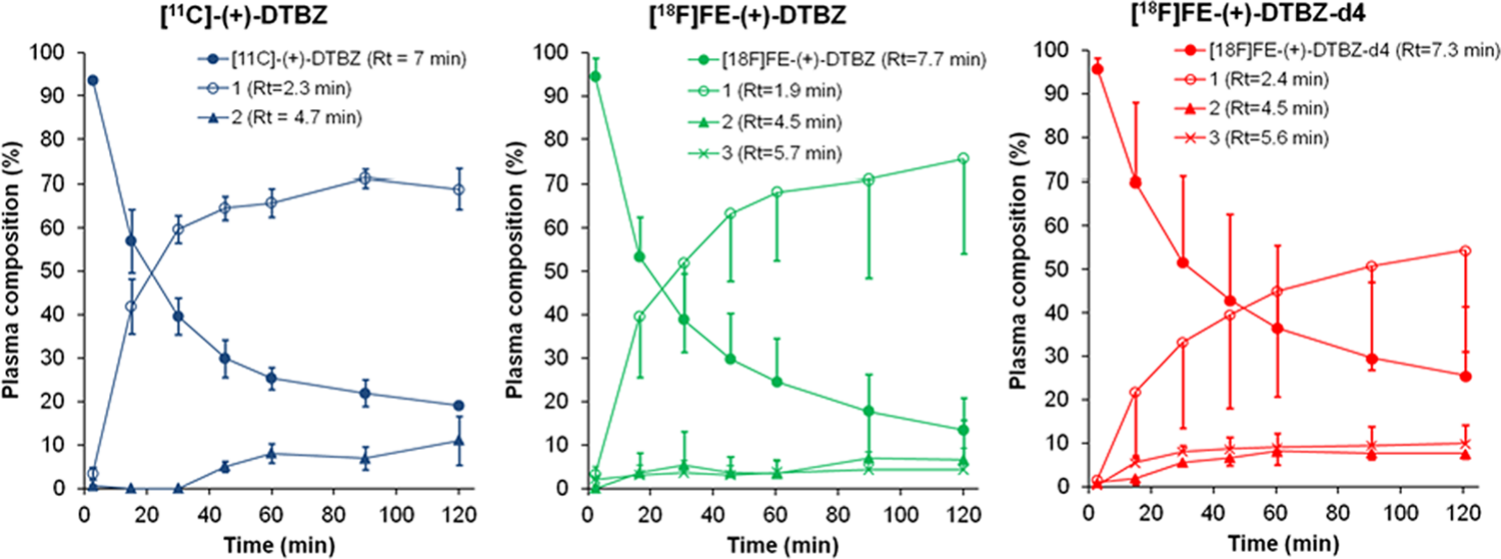
Radiometabolite analysis during the course of the PET measurements.

The radioactivity in venous blood samples, plasma, and remaining protein pellet after deproteinization of plasma with acetonitrile were measured by a well counter. The recovery of radioactivity from plasma into acetonitrile was more than 90%. The plasma obtained from venous blood samples taken at various time points following the injection of radioligands was analyzed by reverse-phase radio-HPLC. All of the detected radiometabolites were less lipophilic than the parent radioligands. For all three radioligands, two detected unidentified radiometabolites were eluted with an Rt of 2.3 and 4.7 min, whereas the parent radioligands were eluted with an Rt over 7 min. In the case of [^18^F]FE-DTBZ and [^18^F]FE-DTBZ-d4, one extra radiometabolite was detected eluting with an Rt of 5.6 min. [^18^F]FE-DTBZ-d4 showed slightly slower radiometabolism. At 90 min after injection of the radioligands, the percent of unchanged [^11^C]DTBZ, [^18^F]FE-DTBZ, and [^18^F]FE-DTBZ-d4 in plasma was 22 ± 3, 10 ± 4, and 30 ± 17%, respectively (Figure 6).

The protein binding of all of the radioligands was measured using ultrafiltration.^28^ The plasma free fraction (*f*_P_) of [^18^F]FE-DTBZ-d4, [^18^F]FE-DTBZ, and [^11^C]DTBZ was 52 ± 1, 56 ± 0, and 47 ± 5%, respectively.

This study was designed specifically to evaluate the *in vivo* properties of [^18^F]FE-DTBZ-d4 as a potential radioligand for imaging and quantification of VMAT2 in the nonhuman primate brain. The kinetic properties and *in vivo* radiometabolism of [^18^F]FE-DTBZ-d4 were compared to the well-established VMAT2 radioligand [^11^C]DTBZ and to the nondeuterated analogue [^18^F]FE-DTBZ.

The results of the study suggest that [^18^F]FE-DTBZ-d4 has better *in vivo* properties than [^11^C]DTBZ and [^18^F]FE-DTBZ, in terms of higher brain uptake, earlier peak equilibrium, and a higher peak-to-late uptake ratio. [^18^F]FE-DTBZ-d4 was also found to be more stable in plasma, likely due to the incorporation of four atoms of deuterium, a procedure known to decrease the *in vivo* metabolism of radioligands.^26^ Finally, the nondisplaceable binding potential (BP_ND_) estimated in key regions of the nigrostriatal system was also found to be higher for [^18^F]FE-DTBZ-d4 as compared with [^11^C]DTBZ and [^18^F]FE-DTBZ ([^18^F]FE-DTBZ-d4 > [^18^F]FE-DTBZ > ^11^C]DTBZ). The *in vivo* binding of [^18^F]FE-DTBZ-d4 to VMAT2 was also confirmed by pretreatment and displacement experiments with the VMAT2 inhibitor tetrabenazine. In both types of experiments, the brain radioactivity of [^18^F]FE-DTBZ-d4 in the striatum and midbrain approached the one measured in reference regions, such as the occipital cortex and the cerebellum. The clear effect of tetrabenazine on the TACs of [^18^F]FE-DTBZ-d4 in the displacement experiment also confirms the reversibility of the binding of the radioligand to VMAT2.

The main purpose of developing a deuterated analogue was to improve *in vivo* stability and to decrease defluorination, thereby decreasing bone uptake. The visual interpretation of [^18^F]FE-DTBZ PET images does not suggest, however, the presence of obvious bone uptake. Therefore, it seems that the main advantages of deuterium incorporation are improved *in vivo* stability and increased brain uptake of [^18^F]FE-DTBZ-d4.

The objective of this study was to compare the three radioligands described above and to evaluate the effects of the incorporation of deuterium on the *in vivo* properties of [^18^F]FE-DTBZ-d4. One limitation is that the established fluoropropyl analogue [^18^F]AV-133 was not included in the comparison. Therefore, we do not know whether [^18^F]FE-DTBZ-d4 has better *in vivo* properties than [^18^F]AV-133 in the nonhuman primate. This evaluation needs a separate head-to-head comparison, which was outside the scope of this study.

## Materials and Methods

### General

Liquid chromatographic (LC) analysis was performed with a Merck-Hitachi gradient pump and a Merck-Hitachi, L-4000 variable wavelength UV detector. The precursor 9-*O-*desmethyl-(+)-DTBZ was purchased from ABX GmbH (Germany). The authentic reference standards FE-(+)-DTBZ-d4, FE-(+)-DTBZ, and DTBZ were purchased from Pharmasynth AB (Estonia). All other chemicals and reagents were purchased from commercial suppliers. Solid-phase extraction (SPE) cartridges SepPak QMA light and SepPak C18 Plus were bought from Waters (Milford, Mass). SepPak QMA light was activated using a K_2_CO_3_ solution (0.5 M, 10 mL) followed by water (15 mL, 18 MΩ). The C18 cartridge was activated using EtOH (10 mL), followed by sterile water (10 mL). Fluorine-18 fluoride was produced at the Karolinska University Hospital (Stockholm, Sweden) from a GEMS PETtrace Cyclotron using 16.4 MeV protons.

### Synthesis of [^11^C]DTBZ

[^11^C]Methyl iodide ([^11^C]CH_3_I) was produced according to a previously published method.^29^ In short, [^11^C]CH_4_ was produced in the cyclotron and collected in a Porapak Q trap cooled with liquid nitrogen. [^11^C]CH_4_ was released from the trap by heating with pressurized air and subsequently mixed with iodine vapors at 60 °C followed by a radical reaction at 720 °C. After the reaction, [^11^C]CH_3_I was collected in a Porapak Q trap at room temperature (RT) and was released by heating at 180 °C. [^11^C]CH_3_OTff was produced by the online transfer of [^11^C]CH_3_I through a glass column packed with silver triflate at 165 °C.

The radiosynthesis of [^11^C]DTBZ was obtained by trapping [^11^C]CH_3_OTf at RT in a reaction vessel containing precursor 9-*O*-desmethyl-(+)-DTBZ (0.4–0.6 mg) and sodium hydroxide (NaOH) (0.5 M, 6 μL) in acetone (300 μL). After the end of trapping, the reaction mixture was diluted with 500 μL of sterile water before injecting into the built-in high-performance liquid chromatography (HPLC) system to purify the labeled compound. The HPLC system was equipped with a semipreparative reverse-phase μ-Bondapak HPLC column (C18, 7.8 Ø × 300 mm, 10 μm, Waters) for purification. The column outlet was coupled with a UV absorbance detector (λ = 254 nm) followed by a GM tube for radioactivity detection. HPLC mobile-phase CH_3_CN/10 mM H_3_PO_4_ (15/85) with a flow rate of 6 mL/min led to a complete separation of [^11^C]DTBZ (retention time = 9 min) from impurities. The radioactive fraction corresponding to pure [^11^C]DTBZ was collected from HPLC and evaporated to dryness followed by formulation in phosphate-buffered saline (6 mL). The formulated product was then sterile filtered through a Millipore Millex GV filter unit (0.22 μm) for further use *in vivo.*

### Synthesis of [^18^F]FE-DTBZ and [^18^F]FE-DTBZ-d4

Synthesis of [^18^F]FE-DTBZ/[^18^F]FE-DTBZ-d4 was done by following a previously published method.^25^ The crude [^18^F]FEtBr/[^18^F]FEtBr-d4 was purified by distillation at 90 °C under nitrogen flow followed by passing through a tube filled with P_2_O_5_ and trapped in a second reaction vessel (1 mL) at −5 °C containing the precursor 9-*O-*desmethyl-(+)-DTBZ (2.0–2.5 mg, 6.55–8.19 μmol) and NaOH (15 μL, 5 M in water) in *N,N*-dimethylformamide (DMF, 500 μL).

The reaction mixture was heated at 110 °C for 5 min to synthesize [^18^F]FE-DTBZ/[^18^F]FE-DTBZ-d4, followed by cooling to RT and was diluted with water to a total volume of 1 mL before injecting into a built-in HPLC system, which was equipped with a semipreparative reverse-phase μ-Bondapak HPLC column (C18, 7.8 Ø × 300 mm, 10 μm, Waters) for purification. The column outlet was coupled with a UV absorbance detector (λ = 254 nm) followed by a GM tube for radioactivity detection. HPLC mobile-phase CH_3_CN/10 mM H_3_PO_4_ (15/85) with a flow rate of 5 mL/min led to a complete separation of pure [^18^F]FE-DTBZ/[^18^F]FE-DTBZ-d4 (retention time = 16 min) from all other impurities. The radioactive fractions corresponding to the desired products were collected and diluted with water (50 mL, 18 MΩ). The resulting mixture was passed through a preconditioned SepPak tC18 plus cartridge. The SepPak cartridge was washed with water (10 mL) and the retained product, [^18^F]FE-DTBZ/[^18^F]FE-DTBZ-d4, was eluted with 1 mL of ethanol in a sterile vial containing a phosphate-buffered saline solution (PBS, 7 mL).

### Quality Control of [^11^C]DTBZ/[^18^F]FE-DTBZ/[^18^F]FE-DTBZ-d4

The radiochemical purity, identity, stability, and molar activity (MA) were analyzed using an HPLC system, which included an ACE RP column (C18, 3.9 Ø × 250 mm, 5 μm particle size), a Merck-Hitatchi L-7100 Pump, and an L-7400 UV absorbance detector (λ = 254 nm) coupled to a radioactive detector (b-flow, Beckman, Fullerton, CA). CH_3_CN/50 mM H_3_PO_4_ (15/85) was used as the HPLC mobile phase with a flow rate of 3 mL/min to elute the product. The effluent was monitored with an UV absorbance detector (ƛ = 254 nm) coupled to a radioactive detector (b-flow, Beckman, Fullerton, CA) and the product was eluted with a retention time (Rt) of 4–5 min for [^18^F]FE-DTBZ/[1^8^F]FE-DTBZ-d4/[^11^C]DTBZ. The identity of all radioligands was confirmed by coinjection with the authentic nonradioactive reference standards.

MA was calibrated for UV absorbance (λ = 254 nm) response per mass of ligand and calculated as the radioactivity of the radioligand (GBq) divided by the amount of the associated carrier substance (μmol). Each sample was analyzed three times and compared to a reference standard.

### PET Measurements in Cynomolgus Monkeys

The Animal Ethics Committee of the Swedish Animal Welfare Agency (Dnr N185/14) approved the study protocol. All of the experiments were performed according to the “Guidelines for planning, conducting and documenting experimental research” (Dnr 4820/06-600) of Karolinska Institutet.^30,31^ The NHPs were housed in the Astrid Fagraeus Laboratory (AFL) of the Swedish Institute for Infectious Disease Control, Solna, Sweden. Anesthesia was induced by intramuscular injection of ketamine hydrochloride (approximately 10 mg/kg, Ketaminol vet. Intervet) and maintained by the administration of a mixture of sevoflurane, oxygen, and medical air after endotracheal intubation. The head was immobilized using a fixation device.^32^

Three female cynomolgus monkeys (NHP1, NHP2, and NHP3) (weight of 5.8–6.95 kg) were studied in 10 different experimental days for a total of 12 PET experiments. All PET experiments were performed using a high-resolution research tomograph (HRRT) PET scanner (Siemens Molecular Imaging, Knoxville, TN). A 6 min transmission scan was performed every time before the injection of the radioligands using a single ^137^Cs source. List-mode data were reconstructed with a series of 35 frames (10 s × 4, 20 s × 4, 1 min × 3, 3 min × 7, and 6 min × 16) for ^11^C and (10 s × 4, 20 s × 4, 1 min × 4, 3 min × 7, 6 min × 16, and 12 min × 5) for ^18^F, using the ordinary Poisson-3D-ordered subset expectation maximization (OP-3D-OSEM) algorithm with 10 iterations and 16 subsets including modeling of the point spread function (PSF), correction for attenuation, random, and scatter. The resolution of the reconstructed images was 1.5 mm in full-width at half maximum.^33^

All three monkeys underwent two PET measurements on the same day at once. The first PET measurement (123 min) was performed with [^11^C]DTBZ (181 ± 7 MBq); the second PET measurement (183 min) was performed with [^18^F]FE-DTBZ-d4 (187 ± 14 MBq), 3 h after the first one. The third PET measurement with [^18^F]FE-DTBZ (183 min) was performed on a separate day. In two monkeys (NHP1 and NHP2), the third PET measurement with [^18^F]FE-DTBZ (180 ± 2 MBq) was performed 65 and 88 days after the first PET measurement. In NHP3, the third PET measurement with [^18^F]FE-DTBZ (199 MBq) was conducted after 201 days of the first PET measurement. In NHP1, one PET measurement was performed after pretreatment with tetrabenazine (2 mg/kg), which was administered 15 min prior to the administration of [^18^F]FE-DTBZ-d4. In NHP2, two displacement experiments were performed with the administration of tetrabenazine (2 mg/kg) 25 min after the injection of [^18^F]FE-DTBZ-d4. Venous blood sampling was performed manually for the measurement of protein binding, blood, and plasma radioactivity and radiometabolite analysis. Blood samples were collected at different time points: 5 min before the injection of the corresponding radioligand followed by 2.5, 15, 30, 45, 60, 90, and 120 min after the injection. In the case of [^18^F]FE-DTBZ-d4 and [^18^F]FE-DTBZ, venous blood sampling was also performed at 180 min after the injection due to the longer half-life of fluorine-18.

The regions of interest (ROIs) were delineated manually on the MRI images of each NHP for putamen, caudate, midbrain, occipital cortex, and cerebellum. The MRI of all of the individual NHPs was coregistered to summed PET images of the whole PET measurement. The time–activity curves of NHP brain regions were generated from dynamic PET data with the application of the coregistration parameters to ROIs. Quantification was performed using the Logan graphical analysis, and the cerebellum was used as a reference region. The outcome measure was the binding potential (BP_ND_).

### Radiometabolite Analysis

Radiometabolite analysis was performed following a method reported elsewhere.^34^ Venous blood samples (2 mL) were obtained from the monkey at different time points such as 2.5, 15, 30, 45, 60, 90, 120, 150, and 180 min after injection of [^18^F]FE-DTBZ/[^18^F]FE-DTBZ-d4. For [^11^C]-DTBZ, venous blood samples (2 mL) were obtained at time points 2.5, 15, 30, 45, 60, and 90 min after injection. Collected blood was centrifuged at 2000*g* for 2 min to obtain the plasma (0.5 mL). The plasma was mixed with 1.4 times volume of acetonitrile followed by centrifugation at 2000*g* for 4 min. The extract was separated from the pellet and was diluted with water before injecting into the HPLC equipped with a reverse-phase μ-Bondapak HPLC column, (300 mm × 10 mm I.D). An Agilent binary pump (Agilent 1200 series) coupled to a manual injection valve (7725i, Rheodyne), 1–3.0 mL loop, and a radiation detector (Oyokoken, S-2493Z) placed in a shield of 50 mm thick lead was used for radiometabolite measurements. Chromatographic software (ChemStation Rev. B.04.03; Agilent) was used for the data collection and control of the LC system. The accumulation time of the radiation detector was 10 s. Chromatographic separation was achieved by gradient elution using acetonitrile (A) and 0.01 M H_3_PO_4_ (B) as the mobile phase with a flow rate 6.0 mL/min, according to the following program: 0–8.0 min, (A/B) 50:50 → 95:5 v/v; 8.0–10.0 min, (A/B) 95:5 v/v. Peaks for radioactive compounds eluting from the column were integrated and their areas were expressed as a percentage of the sum of the areas of all detected radioactive compounds (decay-corrected to the time of injection on the HPLC). To calculate the recovery of radioactivity from the system, an aliquot (2 mL) of the eluate from the HPLC column was measured and divided by the amount of the total injected radioanalyte.

### Plasma Protein Binding

Plasma protein binding of all three radioligands was measured in duplicate in baseline conditions. NHP blood plasma (500 μL) and a PBS solution (pH 7.4, KCl 0.2 mg, KH_2_PO_4_ 0.2 mg, Na_2_HPO_4_ 1.42 mg, NaCl 8 mg in 1 mL) were mixed with the respective radioligand (50 μL, approx. 20 MBq in PBS). The plasma mixture was incubated for 10 min at room temperature and a small portion (20 μL) from each incubation mixture was measured in a well counter. A portion (200 μL) from all individual incubation mixtures was pipetted out into ultrafiltration tubes (Millipore Centrifree YM-30) and centrifuged at 3000 rpm for 15 min. Samples (20 μL) from each filtrate were counted in a well counter. The following formula was used to calculate the protein-bound fraction.
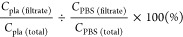
where *C*_pla (total)_ is the radioactivity concentration of the incubation mixture in plasma, *C*_PBS (total)_ is the radioactivity concentration of the incubation mixture in PBS, and *C*_pla (filtrate)_ and *C*_pla (filtrate)_ are the radioactivity concentrations from filtrate samples.

## Conclusions

The radioligand [^18^F]FE-DTBZ-d4 was explored extensively in nonhuman primate brains to visualize VMAT2, which is a potential target for screening PD. The results demonstrated higher brain uptake of [^18^F]FE-DTBZ-d4 with faster washout as well as with increased stability *in vivo* compared to nondeuterated [^18^F]FE-DTZ and [^11^C]DTBZ. The pretreatment and displacement studies verified specificity toward VAMT2 and reversible binding. These results make [^18^F]FE-DTBZ-d4 a suitable radioligand for quantification of VMAT2 in the nonhuman primate brain, with better imaging properties over [^11^C]DTBZ and [^18^F]FE-DTBZ. A preliminary comparison suggests that [^18^F]FE-DTBZ-d4 has potential for further development as a PET radioligand for imaging of binding to VMAT2 in the human brain *in vivo*.
